# Comparative Characterization of Tumor Microenvironments in Monophasic and Biphasic Synovial Sarcomas

**DOI:** 10.3390/ijms262010119

**Published:** 2025-10-17

**Authors:** Anna Kosyreva, Enar Jumaniyazova, Alexandra Sentyabreva, Ekaterina Miroshnichenko, Dzhuliia Dzhalilova, Timur Fetisov, Anastasia Tararykova, Anastasiya Lokhonina, Timur Fatkhudinov

**Affiliations:** 1 Research Institute of Molecular and Cellular Medicine, Peoples’ Friendship University of Russia, 6 Miklukho-Maklaya Street, 117198 Moscow, Russia; 2 Avtsyn Research Institute of Human Morphology, Petrovsky National Research Centre of Surgery, 3 Tsyurupy Street, 117418 Moscow, Russia; 3 N.N. Blokhin National Medical Research Center of Oncology, Ministry of Health of the Russian Federation, 23 Kashirskoe Highway, 115478 Moscow, Russia

**Keywords:** synovial sarcomas, soft-tissue sarcoma, tumor microenvironments, monophasic sarcoma, biphasic sarcoma, tumor-associated macrophages

## Abstract

The impact of histological subtype on immunogenic properties of the tumor microenvironment in synovial sarcomas (SSs) remains understudied. This study aimed to conduct a comparative assessment of tumor microenvironments in monophasic and biphasic SSs. During the study, biomaterial from nine patients with SS was analyzed using IHC analysis, flow cytometry, and real-time PCR. All tumors were infiltrated with CD45+ leukocytes, including the diffusely scattered CD68+ macrophages. FAP+ cells were identified in 7/9 observations, including both monophasic and biphasic tumors. CD4+ T cells and CD20+ B cells were identified by IHC in biphasic SS. The flow cytometry assay revealed significantly higher counts of CD4+ and CD8+ lymphocytes in biphasic SS. IHC revealed E-cadherin expression specifically in the epithelial component of biphasic SS. Vimentin expression in the mesenchymal component of biphasic SS was stronger than in monophasic tumors. The reverse transcription real-time PCR assay revealed higher expression of tumor markers *CDKN2A*, *EGFR*, and *PDGFRL* in monophasic SS. Expression levels of M2 macrophage marker *ARG1* and levels of M1 macrophage marker *NOS2* in monophasic SS were higher than in biphasic tumors. Biphasic and monophasic SSs revealed distinct molecular patterns and differential degrees of T lymphocyte and M2 macrophage infiltration. Biphasic SSs are characterized by the presence of lymphocytes in the tumor, while monophasic SSs show more pronounced infiltration with M2 macrophages. Monophasic tumors are characterized by higher expression of cancer-related genes *CDKN2A*, *EGFR*, and *PDGFRL*, which can be considered as potential targets for treatment. Our study is limited to a small sample of patients. This is due to the rarity of synovial sarcoma, as well as the fact that we recruited patients who had not received radiation or chemotherapy before taking the biomaterial. It was these criteria that made it possible to objectively assess the state of the tumor microenvironment.

## 1. Introduction

Synovial sarcomas (SSs) are malignant soft-tissue tumors of mesenchymal origin with the incidence amounting to 5–10% of primary soft-tissue sarcomas [1]. SS harbor t(X;18) (p11; q11) translocations fusing *SS18* on chromosome 18 to *SSX1*, *SSX2,* or *SSX4* on chromosome X [2]. The *SS18*::*SSX* chimeric oncoproteins disrupt the ATP-dependent chromatin remodeling to activate stem cell-like transcriptional signatures [3].

Histologically, SSs can be divided into monophasic (mesenchymal) and biphasic (mesenchymal and epithelial) subtypes [4]. While both subtypes comprise a core tumor component of spindle-shaped mesenchymal cells, biphasic SSs also have a well-developed epithelial component of heterogeneously differentiated epithelial tumor cells that express epithelial markers, including keratin [5]. Monophasic SSs have no epithelial component. A USA-based study of outcomes in a large clinical cohort reveals slightly better survival among patients with biphasic SSs [6,7]; the finding has not been confirmed in other settings [8,9].

The choice of treatment strategy for SS depends on the particular disease status in terms of malignancy grade and metastasis. The “gold standard” of surgical resection combined with neoadjuvant/adjuvant chemoradiotherapy applies to localized tumor processes; informed decisions on particular treatment schemes should be issued by multidisciplinary boards in reference medical centers, with consideration for malignancy grade, morphological type, risks of metastasis and relapse, and tumor localization/volume. In cases of locally advanced inoperable and/or metastatic disease, the recommendations involve anthracycline-based schemes [10]. Despite the upgrade of anti-tumor drug therapies, 5-year overall survival rates in patients with locally advanced inoperable and/or metastatic SS are ≤10% [11].

In terms of the tumor microenvironment properties, SSs are weakly immunogenic tumors, hence the low rates of response to immune checkpoint inhibitors [12]. While about 80% of SSs express the NY-ESO-1 cancer–testis antigen [13], the counts of tumor-infiltrating T cells to effectuate an immune response are low [14]. Despite developments in immunotherapy for SS, there is limited knowledge regarding differences in cellular composition of the tumor microenvironment between monophasic and biphasic histological subtypes of this malignancy, including the respective balances of tumor-infiltrating lymphocytes and macrophages [15].

Previous studies have positively correlated the tumor-infiltrating CD8+ T cell counts in SS with favorable outcomes, whereas for CD163+ tumor-associated macrophages (TAMs), the corresponding trend is negative [1]. Increased macrophage counts at the boundary with healthy tissues have been associated with poorer outcomes in various high-grade soft-tissue sarcomas [16]. At the same time, TAM counts are known to be highly dynamic and shown to be influenced by multiple factors, including therapies [17]. The impact of histological subtype (monophasic or biphasic) on immunogenic properties of the tumor microenvironment in SS remains understudied [18]. This study aimed to conduct a comparative assessment of tumor microenvironments in monophasic and biphasic SSs.

## 2. Results

### 2.1. Histological Characterization of Biphasic and Monophasic Synovial Sarcomas

**Figure 1:**
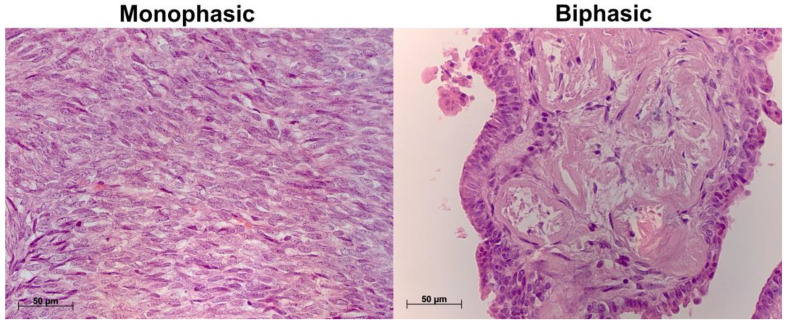
Representative histological images of biphasic and monophasic synovial sarcomas, including a biphasic tumor comprising layer-forming epithelial cells and spindle-shaped mesenchymal cells and a monophasic tumor of spindle-shaped cells growing in multiple directions; stroma was sparse to moderate, containing thin eosinophilic fibers. Hematoxylin and eosin staining, magnification ×640.

Hematoxylin and eosin-stained biphasic SS specimens showed varying proportions of epithelial and spindle-shaped cell components. Epithelial cells were columnar with rounded nuclei and eosinophilic cytoplasm, arranged in layers, while mesenchymal cells were small and spindle-shaped, with a scanty rim of cytoplasm and hyperchromic nuclei (Figure 1). In monophasic SS, the examination revealed spindle-shaped tumor cells only, with atypical nuclei and scanty cytoplasm, growing in multiple directions; stroma was sparse to moderate, containing thin eosinophilic fibers; and there were pre-existing blood vessels with well-formed walls (Figure 1). One of the monophasic SS specimens (n = 7) revealed extensive necrosis.

### 2.2. Immunohistochemistry and Molecular Characterization of Biphasic and Monophasic Sarcomas

**Figure 2:**
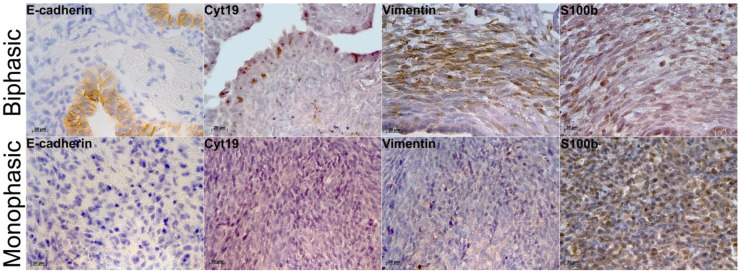
Biphasic (top) and monophasic (bottom) synovial sarcomas stained for E-cadherin (×400), cytokeratin 19 (×640), vimentin (×640), and S100b (×640). Peroxidase reaction immunohistochemistry; nuclei counterstained with hematoxylin.

**Figure 3:**
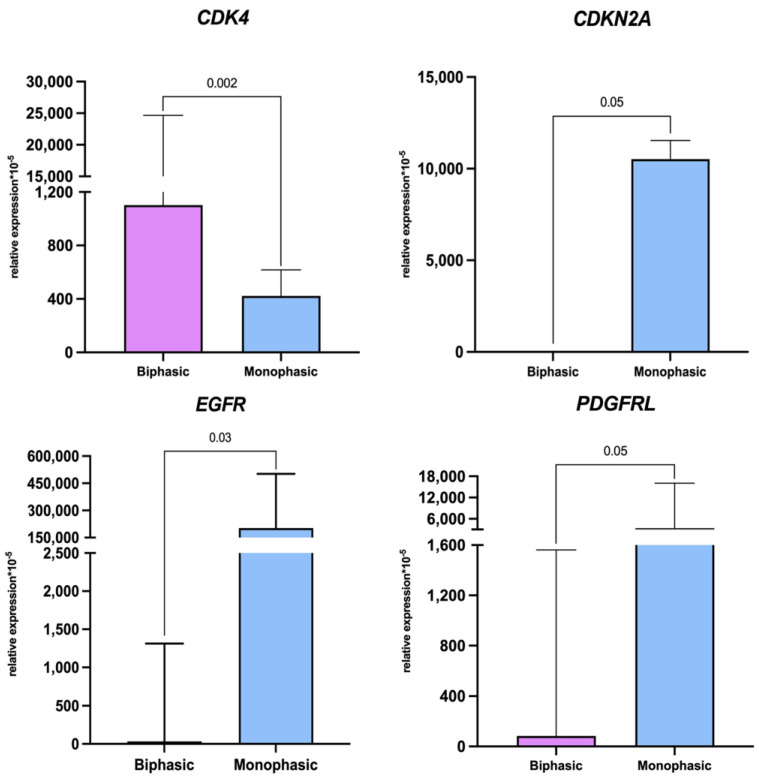
Relative levels of CDKN2A, EGFR, PDGFRL, and *CDK4* mRNA in biphasic (purple, on the left) and monophasic (blue, on the right) synovial sarcomas. Data visualized as median and interquartile range. Mann–Whitney U test.

IHC revealed E-cadherin expression specifically in the epithelial component of biphasic SS (Figure 2). In monophasic SS, the reactions were totally negative (Figure 2), except for one tumor with solitary positive cells. Similarly, the epithelial component of biphasic SS expressed cytokeratin 19 (Figure 2), whereas in monophasic SS, the reactions were negative (Figure 2). Notably, vimentin expression in the mesenchymal component of biphasic SS was stronger than in monophasic tumors (Figure 2). S100b-positive cells were detected in both biphasic and monophasic SSs (Figure 2). The reverse transcription real-time PCR assay revealed higher expression of tumor markers *CDKN2A*, *EGFR*, and *PDGFRL* in monophasic SS compared to biphasic tumors (Table 1, Figure 3).

### 2.3. Tumor Microenvironments in Biphasic and Monophasic Synovial Sarcomas

**Figure 4:**
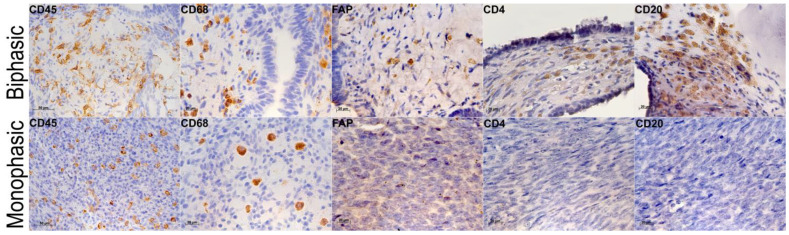
Biphasic (**top**) and monophasic (**bottom**) synovial sarcomas stained for immune cell markers CD45 (×400), CD68 (×640), FAP (×640), CD4 (×640), and CD20 (×400). Peroxidase reaction immunohistochemistry; nuclei counterstained with hematoxylin.

**Figure 5:**
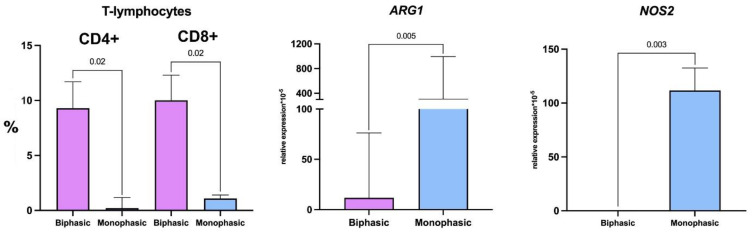
Relative counts of CD4+ and CD8+ T cells in biphasic (purple, on the left) and monophasic (blue, on the right) synovial sarcomas. Data visualized as median and interquartile range. Mann–Whitney U test.

All tumors were infiltrated with CD45+ leukocytes (Figure 4), including the diffusely scattered CD68+ macrophages (Figure 4). In the unique monophasic SS sample with necrosis, CD68+ cells were found in necrotic zones and absent amidst tumor cells. FAP+ cells were identified in 7/9 observations, including both monophasic and biphasic tumors (Figure 4). CD4+ T cells and CD20+ B cells were identified by IHC in biphasic SS. The flow cytometry assay revealed significantly higher counts of CD4+ and CD8+ lymphocytes in biphasic SS compared to monophasic tumors (Figure 5, Table 2), but there were no significant between-group differences in monocyte or macrophage counts. However, the analysis revealed a tendency towards increased CD16+ monocyte content in biphasic SS and, conversely, increased relative counts of the M2 macrophage marker CD206-positive cells in monophasic SS (Table 2).

Expression levels of M2 macrophage marker *ARG1* in monophasic SS were higher than in biphasic tumors (Figure 5, Table 3), which is consistent with the flow cytometry data. Expression levels of M1 macrophage marker *NOS2* were also higher in monophasic SS compared with biphasic tumors (Figure 5, Table 3).

## 3. Discussion

The results indicate high expression levels of E-cadherin and cytokeratin 19 in epithelial cells and stronger reactions for vimentin and S100b in spindle cells of biphasic synovial sarcomas. Microenvironments of biphasic synovial sarcomas are rich in lymphocytes (with both T and B cells identifiable), whereas in monophasic tumors, macrophages predominate and lymphocytes are absent.

Synovial sarcomas (SSs) reveal diffuse expression of the apoptosis regulator BCL2 and often express a transmembrane glycoprotein CD99 (detected in >60% of the cases) [19]. In addition, most of the tumors express transcriptional corepressor TLE1, which is commonly used as a differential diagnosis marker for SS [19] along with NY-ESO-1 [13].

Our analysis of morphological, immunohistochemical, and molecular patterns in SS with regard to histological subtype reveals epithelial positivity for E-cadherin and cytokeratin 19 as well as vimentin and S100b protein expression, the lack of CDKN2A mRNA, and low levels of EGFR and PDGFRL mRNA in biphasic SS. By contrast, monophasic SS typically express *EGFR, PDGFRL*, and (often) high levels of *CDKN2A* but react negatively to epithelial IHC markers.

The negative staining for E-cadherin, a marker of cellular adhesion and epithelial–mesenchymal transition, is consistent with previous findings; in some SS, it has been associated with mutations in the corresponding gene *CDH1* [20]. Cytokeratin 19 expressed in monophasic SS can provide an additional trait for differential diagnosis with peripheral nerve sheath malignancies [21]. In our setting, we observed positive IHC reactions for cytokeratin 19 in the epithelial cells of biphasic SS but not in monophasic tumors.

Vimentin, the principal intermediate filament protein in cells of mesodermal origin, is ubiquitously found in SS [22]. Vimentin translocation to the surface of tumor cells, particularly in circulating tumor elements, has been associated with high metastatic capacity [23]. In our setting, biphasic SS expressed vimentin stronger than monophasic tumors, which may indicate a higher metastatic potential.

S100b protein is expressed in glial cells, melanocytes, adipocytes, and chondrocytes. About 30% of SSs are S100-positive [24,25]. Transcriptomically, SSs clusterize with peripheral nerve sheath malignancies, which may indicate neuromesenchymal origin [26,27]. The involvement of neural crest derivatives in SS is further supported by strong expression of genes encoding the (neuro)cartilage marker collagen IX, neurofilament heavy polypeptide NEFH involved in axonal scaffolding and radial growth, endothelin 3 required for melanocyte and enteric neuron differentiation, and the chondrogenic transcription activator SOX9 [28]. Neural crest precursors have also been implicated in neuroblastoma [29], melanoma [30], and Ewing sarcoma [31] (a family of bone and soft-tissue cancers with EWS::FLI1 or EWS::ERG rearrangements [32]). The expression of S100 protein in both biphasic and monophasic SSs is consistent with the neural origin hypothesis.

CDKN2A/B deletions and correspondingly low levels of CDKN2A mRNA have been reported in high-grade endometrial sarcomas [33] and pediatric sarcomas with BCOR genetic alterations [34]. Notably, CDKN2A/B homo- and heterozygous losses were correlated with decreased overall survival rates in BCOR-altered pediatric soft-tissue sarcomas [34]. The differential expression of CDKN2A with regard to histological subtype in SS (low in biphasic tumors) may also correlate with the prognosis.

The epidermal growth factor receptor (EGFR) is frequently expressed by solid tumors. EGFR is a member of the ErbB family of receptor tyrosine kinases that regulate cell proliferation, survival, adhesion, migration, and differentiation [35]; in certain tumor types, its expression has been associated with poor clinical outcomes [36]. *EGFR* gene expression is more common in SS compared with other sarcomas [37]. In our setting, EGFR mRNA was detected in both biphasic and monophasic SSs but at higher levels in monophasic tumors.

The PDGF receptor family participates in the regulation of connective tissue cell functionalities, including those associated with survival and growth [38]. High expression of PDGF in gastric adenocarcinoma [39], glioma [40], medulloblastoma [41], and other solid malignancies makes it a putative therapeutic target. High PDGFA levels correlate with poor survival rates in high-grade carcinomas [42]. PDGFRL, a specific PDGFRβ agonist, has been suggested to exert a tumor-suppressive effect. Compared with a gastrointestinal stromal tumor, myxoid liposarcoma, and sarcomas with complex genomics, *PDGFRL* expression rates in SS are low; however, its levels have not been analyzed with regard to histological subtype [43]. Our data indicate increased PDGFRL mRNA levels in monophasic SS, which may correlate with the prognosis.

The interaction between PDL1 expressed by tumor cells and its PD-1 receptor on activated T cells leads to suppression of T cells and inhibition of the anti-tumor immune response. PD-L1 expression in tumors is associated with unfavorable outcomes in various solid tumors [44]. In the Bertucci et al. study, PDL1 expression was evaluated in 758 previously untreated STS samples, and the study demonstrated that high expression is an independent unfavorable prognostic factor for metastatic recurrence [45]. In our study, we found PDL1 expression in both types of synovial sarcomas; it was quite high, which coincides with the results of colleagues whose study showed PDL1 expression present in most cases of synovial sarcoma. Preliminary data indicates that it is a prognostic marker associated with adverse outcomes. Thus, PD-L1 can be considered as a potential therapeutic target in patients with synovial sarcoma.

CDK4 is a serine/threonine protein kinase whose activity mediates cell cycle progression through the G1-S phase in preparation for DNA synthesis. In human malignancies, CDK4 associates with cyclin D and regulates the cell cycle through hyperphosphorylation and inactivation of the tumor suppressor protein retinoblastoma (Rb) [46]. A study by Li et al. demonstrated high expression of CDK4 in synovial sarcomas [47]. This suggests that CDK4 may play an important role in the pathogenesis of synovial sarcoma. Furthermore, the researchers noted a link between *CDK4* expression and tumor clinicopathological characteristics: CDK4 expression significantly correlated with higher clinical stage and higher TNM grade in patients with synovial sarcoma, as well as with a worse clinical prognosis in patients with sarcoma [47]. In our study, *CDK4* expression was predominant in biphasic sarcoma samples.

Immune microenvironments are closely involved in tumor progression and response to anti-tumor therapy, strongly influencing the prognosis and clinical outcomes. SSs and other soft-tissue sarcomas are considered “cold tumors”, i.e., with low rates of immunogenic infiltration [48]. However, despite the overall sparsity of elements, the specific composition of infiltrating lymphocytes and TAMs in biphasic and monophasic SSs can be revealing in terms of prognostic significance and may facilitate the development of new treatment approaches. Particular subsets of immune cells may play dual roles, exerting both tumor-promoting and tumor-suppressive effects in the rich context of the tumor microenvironment [49]. It is important to assess the microenvironment-mediated immunological balances in different subtypes of SS to understand the mechanisms of immune evasion and resistance to therapy [50,51].

The results indicate that lymphocytes are well represented in microenvironments of biphasic SS and absent in monophasic SS. High densities of tumor-infiltrating lymphocytes, especially cytotoxic ones, correlate with milder clinical courses and higher survival rates [52]. We demonstrate that biphasic SS contains a variety of lymphocyte subsets (CD4+ helper T cells, CD8+ cytotoxic T cells, and B cells), which may justify the use of neoadjuvant chemotherapy and/or immunotherapy specifically for biphasic SS.

Macrophages are known to provide a dominant regulatory influence in various tumor microenvironments and can be roughly subdivided into two major subsets: anti-tumor (pro-inflammatory) M1 macrophages and pro-tumor (anti-inflammatory) M2 macrophages [53]. M2 macrophages augment tumor progression by producing factors that induce angiogenesis, inhibit the anti-tumor immune responses, and activate tumor cell proliferation and metastasis [54]. CD163 and CD206 are M2 macrophage markers; accordingly, high counts of infiltrating CD163+ cells appear prognostically unfavorable [1]. In our setting, both monophasic and biphasic SSs were characterized by a high content of CD68+ macrophages, including those expressing the M2 phenotype markers CD163 and CD206. It should be noted that, despite the lack of statistically significant differences, relative CD163+ and CD206+ cell counts tended to be higher in monophasic SS compared with biphasic tumors. In addition, monophasic SS revealed higher mRNA levels of both the M1 macrophage marker NOS2 and the M2 macrophage marker *ARG1*, which supports the assumption of stronger immunosuppression in monophasic SS and suggests a poorer response of monophasic SS to chemotherapy as compared with biphasic tumors.

Circulating monocytes participate in tumor progression by migrating from vessels to the site of inflammation, infiltrating tumors, and differentiating into dendritic cells or macrophages [55,56,57]. In osteosarcoma, CD16+ monocytes/macrophages secrete chemokines CCL2, CCL3, and CCL8 to enhance tumor infiltration by immune cells [58]. The more pronounced infiltration by CD16+ monocytes observed by us in biphasic SS can be associated with the higher rates of T and B lymphocyte infiltration compared to monophasic tumors.

The role of immunotherapy in the treatment of STS remains controversial, and further research is needed to better understand its potential benefits in individual cases. Evidence that a limited number of sarcoma patients in each histological subtype of STS experience clinical benefits from ICI treatment may be related to the genetic and immunological heterogeneity that dominates each individual histological subgroup. These data emphasize the importance of in-depth study of the molecular features associated with evading immune surveillance, as well as a targeted study of the microenvironment of different subtypes of soft-tissue sarcomas, in order to create a database of candidate tumors for immunotherapy [59].

## 4. Materials and Methods

### 4.1. Characteristics of Patients Whose Material Was Used in the Study

The study enrolled individuals meeting all of the following criteria:

- Male or female over 18 years of age;
- Morphologically verified diagnosis of synovial sarcoma of soft tissues, *SS18*::*SSX* translocation-positive;
- Karnofsky index ≥ 70;
- No unhealthy habits;
- No chronic diseases in acute/decompensated phase;
- No aggravated oncological history;
- No previous history of anti-tumor medications and radiation therapy.

All patients signed a voluntary informed consent to participate in the study. The data are summarized in Table 4.

Tumor biopsies ≥ 3–5 mm^3^ were collected at the Federal State Budgetary Institution “N.N. Blokhin National Medical Research Center of Oncology” of the Ministry of Health of the Russian Federation. Fresh specimens were placed individually in sterile containers with transport medium (DMEM/F12 with penicillin–streptomycin, 4 °C) and delivered to the laboratory in an insulated box with cooling packs within 8 h.

Our study is limited to a small sample of patients. This is due to the rarity of synovial sarcoma, as well as the fact that we recruited patients who had not received radiation or chemotherapy before taking the biomaterial. These criteria made it possible to objectively assess the state of the tumor microenvironment.

### 4.2. Morphological Characterization of the Tumors: Immunohistochemistry

Tumor biopsies were fixed in 10% buffered formalin (BioVitrum, Saint Petersburg, Russia). After that, the specimens were dehydrated with ethanol of increasing concentration, cleared with xylene, infiltrated with a histological wax, and embedded in paraffin blocks for further slicing (5 µm thick). Histological sections of SSs were stained with hematoxylin and eosin (BioVitrum, Saint Petersburg, Russia).

For IHC, deparaffinized histological sections were unmasked in citrate buffer pH 6.0 with 0.5% Tween-20 at 100 °C and washed in phosphate-buffered saline of pH 7.2. Endogenous peroxidase was blocked with 3% H_2_O_2_; the slides were subsequently incubated in protein buffer (phosphate-buffered saline with 0.1% bovine serum albumin) at room temperature for 30 min to minimize non-specific antibody binding. The immunohistochemistry panel included fibroblast activation protein (FAP) (ab28246, Abcam, Cambridge, UK), CD4 for T lymphocytes (anti-rabbit, Cell Marque, 104R-26, 1:100; Rocklin, CA, USA), and CD20 for B lymphocytes (anti-mouse, Cell Marque, 120M-86, 1:50; Rocklin, CA, USA) primary antibodies topped with secondary caprine anti-rabbit IgG HRP (SAA544Rb19, CloudClone; Houston, TX, USA) or goat anti-mouse HRP (ab6789; Abcam, Cambridge, UK). The peroxidase reactions were developed with 3,3′-diaminobenzidine (DAB) as a substrate.

An additional panel of antibodies was used with a Bond-III Automated IHC Staining System (Leica Biosystems Melbourne Pty Ltd., Melbourne, Australia) to study tumor microenvironments; the targets included the CD68 (514H12) macrophage marker, E-cadherin cell adhesion protein, and the CD45 common leukocyte antigen.

The IHC study was carried out at a qualitative level, noting the presence or absence of the protein being studied in the tumor.

### 4.3. Flow Cytometry

Homogenized tumor tissue samples with a cell count of 106 per mL were analyzed by flow cytometry to determine absolute counts of CD14+ and CD16+ monocytes, CD45+ leukocytes, CD68+ macrophages, CD86+ M1 macrophages, CD163+ and CD206+ M2 macrophages, and CD4+ and CD8+ lymphocytes. The counts were performed in a MACSQuant^®^ Analyzer (Miltenyi Biotec, Bergisch Gladbach, Germany) using the following antibodies: anti-human CD4-FITC, anti-human CD163-APC, anti-human CD16-PE, anti-human CD68-PE-Vio-770, anti-human CD45-VioBlue, anti-human CD14-FITC, anti-human CD86-PE, and anti-human CD206-PerCPVio700, all by Miltenyi Biotec.

### 4.4. Reverse Transcription Real-Time Polymerase Chain Reaction Assay

To preserve RNA, the specimens were placed in 1 mL of RNAlater Stabilization Solution (QIAGEN, Hilden, Germany), incubated 24 h at 4 °C, and stored at −70 °C. Total RNA was isolated using the RNeasy Plus Mini Kit (QIAGEN, Hilden, Germany) and stored at −70 °C. First-strand complementary DNA was synthesized using the MMLV RT Kit (Evrogen, Moscow, Russia) in accordance with the manual and stored at −70 °C. Polymerase chain reactions (PCR) were set up with 5X qPCRmix-HS SYBR PCR (Evrogen, Moscow, Russia; containing SYBR Green I intercalating dye to enable real-time fluorescence detection) and run in a DTprime real-time PCR instrument (DNA-Technology, Protvino, Russia). Levels of ARG1, NOS2, CDKN2A, EGFR, and PDGFRL mRNA were calculated relative to corresponding GAPDH mRNA levels as a reference using the following formula: [A]0/[B]0 = EΔC(T), where [A]0 and [B]0 are initial concentrations of the gene of interest and GAPDH mRNA in the PCR mixture, respectively; E is reaction efficiency (taken as 1.98); and ΔC(T) is the threshold cycle difference for GAPDH and the gene of interest. The oligonucleotide primers were designed in Primer-BLAST software 8.0 (NCBI) and custom-produced by Evrogen (Moscow, Russia); the structures are given in Table 5.

### 4.5. Statistics

Numerical data distributions were assessed using the Kolmogorov–Smirnov test. Considering the non-normality of the distributions, the non-parametric Mann–Whitney U test (Statistica 8.0) was applied to compare the groups; the differences were considered statistically significant at *p* ≤ 0.05. The data are presented as median and interquartile range, Med (25%; 75%), and shown graphically using span diagrams with designated median, interquartile range, and lower and upper extremes built in GraphPad Prism 8.0.

## 5. Conclusions

Biphasic and monophasic SSs are histological subtypes of a highly prevalent cancer entity. In our setting, biphasic and monophasic SSs revealed distinct molecular patterns and differential degrees of T lymphocyte and M2 macrophage infiltration. Biphasic SSs are characterized by the presence of lymphocytes (both T and B cells) in the tumor, while monophasic SSs show more pronounced infiltration with M2 macrophages. Apart from the more immunosuppressive microenvironment, monophasic tumors are characterized by higher expression of cancer-related genes *CDKN2A*, *EGFR,* and *PDGFRL*, which can be considered as potential targets for treatment.

## Figures and Tables

**Table 1:** Relative levels of CDKN2A, EGFR, PDGFRL, PDL1, and CDK4 mRNA in biphasic and monophasic synovial sarcomas.

Gene	Biphasic (n = 2) ×10^−5^	Monophasic (n = 7) ×10^−5^	*p*-Value
*CDKN2A*	0 (0–0)	10,520 (9803–11,238)	0.05
*EGFR*	188.0 (32.3–2315)	243,033 (161,312–392,040)	0.03
*PDGFRL*	694 (84.2–1903.8)	208,580,433 (26,171,059–1,576,930,097)	0.05
*PDL1*	3069 (897.4–3069)	1979 (913.3–5795)	0.9
*CDK4*	1102 (867.8–24,668)	423.3 (262.4–617.4)	0.002

**Table 2:** Relative counts of leukocytes, monocytes, M1 and M2 macrophages, and T cells in biphasic and monophasic synovial sarcomas.

Cell Type/Marker(s)		Biphasic (n = 2)	Monophasic (n = 7)	*p*-Value
Leukocytes	CD45+	33.5 (13.5–53.5)	21.6 (9.7–33.0)	
Monocytes	CD14+	10.0 (4.6–15.3)	9.7 (7.7–24.1)	
	CD16+	32.7 (5.2–60.3)	3.7 (1.9–13.9)	
Macrophages	CD68+	38.6 (17.6–59.6)	56.4 (30.8–69.0)	
	CD86+ M1	12.9 (5.9–19.9)	25.0 (18.7–39.31)	
	CD163+ M2	27.9 (9.8–46.0)	49.7 (32.2–53.8)	
	CD206+ M2	19.4 (15.1–23.6)	44.8 (32.2–57.3)	
T cells	CD4+ helpers	9.3 (6.9–11.7)	0.2 (0.08–1.2)	0.04
	CD8+ cytotoxic	10.0 (7.8–12.3)	1.1 (0.6–1.4)	0.04

**Table 3:** Relative mRNA levels for macrophage markers in biphasic and monophasic synovial sarcomas.

		Biphasic (n = 2) ×10^−5^	Monophasic (n = 7) ×10^−5^	*p*-Value
Macrophage markers	*NOS2* (M1)	0 (0–0.4)	112 (28–133)	0.008
	*ARG1* (M2)	22.2 (5.9–76.2)	311 (229–994)	0.01

**Table 4:** Patient data.

Parameter	n (%)
Total number of patients	9 (100)
*Age at diagnosis, years*	
<20	2 (22)
≥20	7 (78)
*Sex*	
Male	4 (44)
Female	5 (66)
*Localization*	
Upper limbs	0 (0)
Lower limbs	7 (78)
Torso	2 (22)
Head and neck	0 (0)
*Disease episode*	
Newly diagnosed tumor	6
Relapse	3
*Size of tumor node,* cm	
≤5	0 (0)
>5	7 (78)
>10	1 (11)
>15	1 (11)
*Malignancy grade*	
I	0 (0)
II	0 (0)
III	9 (100)
IV	0 (0)
*Presence of distant metastases*	
No	7 (78)
Yes	2 (22)
*Histological subtype*	
Monophasic sarcoma	7 (78)
Biphasic sarcoma	2 (22)

**Table 5:** Oligonucleotide primer sequences.

Gene	Primer	Nucleotide Sequence
*ARG1*	Forward	AAA GGG ACA GCC ACG AGG AG
	Reverse	GGA TGT CAG CAA AGG GCA GG
*NOS2*	Forward	TGC TTT GTG CGG AAT GCC AG
	Reverse	ATG TGG TCC TCA TCT GGG CG
*CDKN2A*	Forward	AGT TAC GGT CGG AGG CCG AT
	Reverse	TGG TTA CTG CCT CTG GTG CC
*EGFR*	Forward	CCC CCT GAC TCC GTC CAG TA
	Reverse	CCC AAC TGC GTG AGC TTG TT
*PDGFRL*	Forward	GCT ACC CTG CGT ATC TGG AC
	Reverse	ATT CAC CTG TGT CTG CCG AG
*PDL1*	Forward	CCT TTG CCT CCA CTC AAT G
	Reverse	AAC AGG GTG GTT ACA GCG AT
*CDK4*	Forward	GTT CGT GAG GTG GCT TTA CTG
	Reverse	CCA ACT GGT CGG CTT CAG
*GAPDH*	Forward	TGG TGA AGA CGC CAG TGG A
	Reverse	GCA CCG TCA AGG CTG AGA AC

## Data Availability

The original contributions presented in this study are included in the article. Further inquiries can be directed to the corresponding author.

## Abbreviations

The following abbreviations are used in this manuscript:

SS	Synovial sarcomas
TAM	Tumor-associated macrophage
FAP	Fibroblast activation protein
EGFR	Epidermal growth factor receptor
PDGFRL	Platelet-derived growth factor receptor-like
CDKN2A	Cyclin-dependent kinase inhibitor 2A
PDL1	Programmed death ligand 1
IHC	Immunohistochemistry
ICI	Immune checkpoint inhibitors
